# Polarization and angular insensitive bendable metamaterial absorber for UV to NIR range

**DOI:** 10.1038/s41598-022-08829-2

**Published:** 2022-03-22

**Authors:** Md Mizan Kabir Shuvo, Md Imran Hossain, Sultan Mahmud, Sydur Rahman, Md Tajmiul Hasan Topu, Ahasanul Hoque, Sikder Sunbeam Islam, Mohammad S. Soliman, Sami H. A. Almalki, Md. Shabiul Islam, Mohammad Tariqul Islam

**Affiliations:** 1 Department of Electrical and Electronic Engineering, Mymensingh Engineering College, University of Dhaka, Dhaka, Bangladesh; 2 Department of Electrical and Electronic Engineering, International Islamic University Chittagong, Chittagong, Bangladesh; 3 Department of Electrical, Electronic and Systems Engineering, Faculty of Engineering and Built Environment, Universiti Kebangsaan Malaysia, 43600 Bangi, Malaysia; 4 Department of Electrical and Electronic Engineering, Bangladesh University of Engineering and Technology, Dhaka, 1205 Bangladesh; 5 Institute of Climate Change, Universiti Kebangsaan Malaysia, 43600 Bangi, Malaysia; 6 Department of Electrical Engineering, Faculty of Energy Engineering, Aswan University, Aswan, 81528 Egypt; 7 Department of Electrical Engineering, College of Engineering, Taif University, P.O. Box 11099, Taif, 21944 Kingdom of Saudi Arabia; 8 Faculty of Engineering, Multimedia University, Persiaran Multimedia, 63100 Cyberjaya, Selangor Malaysia; 9 Electrical Engineering Department, College of Engineering, University of Ha’il, Ha’il, 81481 Saudi Arabia

**Keywords:** Materials science, Condensed-matter physics, Materials for optics, Nanoscale materials, Optics and photonics, Optical materials and structures, Energy science and technology, Energy harvesting, Nanoscience and technology, Nanoscale devices

## Abstract

Broadband absorbers are required for solar energy harvesting because they efficiently absorb the incident photon in the wide-ranging solar spectrum. To ensure high absorption of photons, metamaterial absorbers (MMAs) have been a growing area of interest in recent years. In this article, an MMA is proposed using a metal–insulator–metal (MIM) structure (Ni–SiO_2_–Ni) that shows a near-unity broadband absorption of wavelengths from 300 to 1600 nm, with a 95.77% average absorption and a peak absorption of 99.999% at 772.82 nm. The MMA is polarization insensitive as well as wide incident angle stable. Analysis of the effects of mechanical bending on the absorption of the proposed structure shows that absorption holds satisfactory values at different degrees of mechanical loading. The suggested MMA unit cell structure was computationally simulated using the Finite Integration Technique (FIT) and verified using the Finite Element Method (FEM). To analyze the feasibility of the proposed MMA as a solar cell, it is investigated with the universal AM 1.5 solar spectrum characteristics. Besides solar energy harvesting, the proposed MMA unit cell may be employed in a variety of diverse optical applications, including sensors, detectors, and imaging.

## Introduction

In today's world, solar radiation is the most reliable form of renewable energy available. In terms of solar radiation, the light detected on Earth is nearly 7.5% ultraviolet, 48% visible, and 43% infrared radiations^1^. Therefore, researchers are focusing their efforts on producing highly efficient solar absorbers that are simple to produce. Metamaterial absorbers (MMAs) are one such type of absorbers as they can be utilized as ultra-broadband solar absorbers. Metamaterial is an engineered structure that possesses distinct electromagnetic properties with negative permittivity as well as negative permeability^2^. First conceptualized in 1968 by V.G. Veselago^3^, metamaterial is used in numerous engineering applications, including perfect lens^4^, optical cloaking^5^, antennas^6,7^, filters^8^, holograms^9^, thermal imaging^10^, sensors^11–14^, and absorbers^15–17^. A meta surface-based absorber is an appealing area of application for metamaterial. MMAs were first introduced by Landy et al., who created an impeccably engineered MMA in 2008^18^. Since then, different kinds of MMAs have been enrolled in numerous electromagnetic wavebands including narrowband^19,20^, broadband^21–23^, polarization sensitive^24^, and polarization insensitive^25,26^. A metamaterial-based absorber comes with a 3-layer structure of metal–insulator–metal (MIM), with the top-most metal called resonator. EM waves are concluded by the ground metal layer of the structure. The dielectric layer between the metal film and the resonator plate assists the structure in creating coupling capacitance^27,28^. The reflection of the incident wave is at a minimum if the metasurface impedance and the open space impedance are adequately matched^29^.

A nanostructure made of Ti-SiO_2_-Al used as a polarization-insensitive MMA in the visible to near-infrared region was reported to have an average absorption of over 90% from 354 to 1066 nm^30^. In this article^31^, a wideband MMA based on a Ti resonator was reported to reach an average absorption of up to 91.4% from 485 to 1495 nm, with an absorption peak of 97.5%. Article^32^ dealt with a cylindrical resonator-based solar absorber composed of Au and SiO_2_ that had a mean absorption of 80.24% in the infrared range. An ultra-wideband MMA with a nanodisk shaped resonator made of Ti gave a mean absorption of 94% in the visible and near-infrared range^33^. In the absorption band of 960 nm from visible to near-infrared, a single-layer MMA presented an average absorption of more than 90%^34^. A four-layer ultra-broadband Ti resonator-based solar radiation absorber working in the UV to NIR range demonstrated an average absorption of 92.7%^35^. This article^36^ studied a multiband broadband absorber consisting of Ag and SU-8 photoresist material that had an average absorption of 80.4% in the range of 400 nm to 1500 nm. This study^37^ presented a theoretical design for a near-ideal solar thermal absorber made of W and SiO_2_ in the range of 300 nm to 2000 nm that had a mean absorption of 91.7% and was unaffected by the polarization or angle of incidence. It can, therefore, be said that polarization insensitivity, incident angle stability, good absorption levels, and ultra-wideband are the key factors to developing a perfect ultra-wideband MMA, a result that is difficult to obtain.

In this article, an ultra-broadband, mechanically stress able, UV to NIR radiation-detaining metamaterial-inspired solar absorber is reported with improved performance. The proposed MMA has a symmetrically balanced structure made of Ni and SiO_2_, which have a higher level of thermal independence. A numerical study on the proposed absorber shows that it has an average absorption of 95.77% from 300 to 1600 nm, an absorption level in the defined region of more than 91.27%, and a peak absorption of 99.99% at 772.82 nm. The suggested MMA does have a near-unity absorption of 99% from 621.84 to 962.12 nm. Furthermore, under AM 1.5 solar irradiation, the calculated average absorption from 300 to 1600 nm is greater than 95%. The proposed MMA is insensitive to incoming wave polarization and demonstrates incident angle stability up to 70° with considerable absorption efficiency. The proposed MMA’s compact symmetric design, considerable mechanical stress, temperature tolerance capability, and high absorption efficiency across an ultra-broadband range distinguish this absorber from other broadband absorbers.

## Materials choice and design procedure

### Materials choice

The proposed nano-absorber design is an MIM structure. For the front resonator as well as ground slab, nickel (Ni-lossy metal) is chosen. Silicon dioxide (SiO_2_-optical) is selected as the dielectric insulator. According to the Computer Simulation Technology (CST) material database for material properties, the refractive indices for Ni and SiO_2_ are 2.16 and 1.45, respectively, at 772.82 nm. The refractive indices are wavelength-dependent. The reason for choosing Ni as the metallic part of the structure lies in its excellent material characteristics. Ni has very high-temperature resistance and corrosion resistance, very low production expense, and simplicity in production^38^. Ni’s autologous behavior, very high melting temperature (∼ 1453 °C), as well as minimal emissivity over longer wavelengths also help the design achieve satisfactory results^39^. The reason for selecting SiO_2_ as an insulating spacer lies in its lossless characteristics in the desired wavelengths^40^. SiO_2_ exhibits high-temperature stability due to its high melting point (∼ 1600 °C). SiO_2_ also exhibits a comparatively negative real part of permittivity rather than a high non-real part of the dielectric constant at the visible spectrum^41^. Consequently, in a breakdown state, the real component of permittivity diminishes, resulting in a more closed propagating wave for an evanescent wave characteristic. This anisotropic tendency also contributes to polarization and propagation regulation within the substrate since the birefringence characteristics of dispersion relation pairs with SiO_2_’s refractive index. Inductance, as well as coupling capacitance, are maintained at a suitable state for this dielectric material. In addition, the proposed structure can withstand high temperatures as the melting point of both materials are significantly very high.

### Design procedure

**Figure 1:**
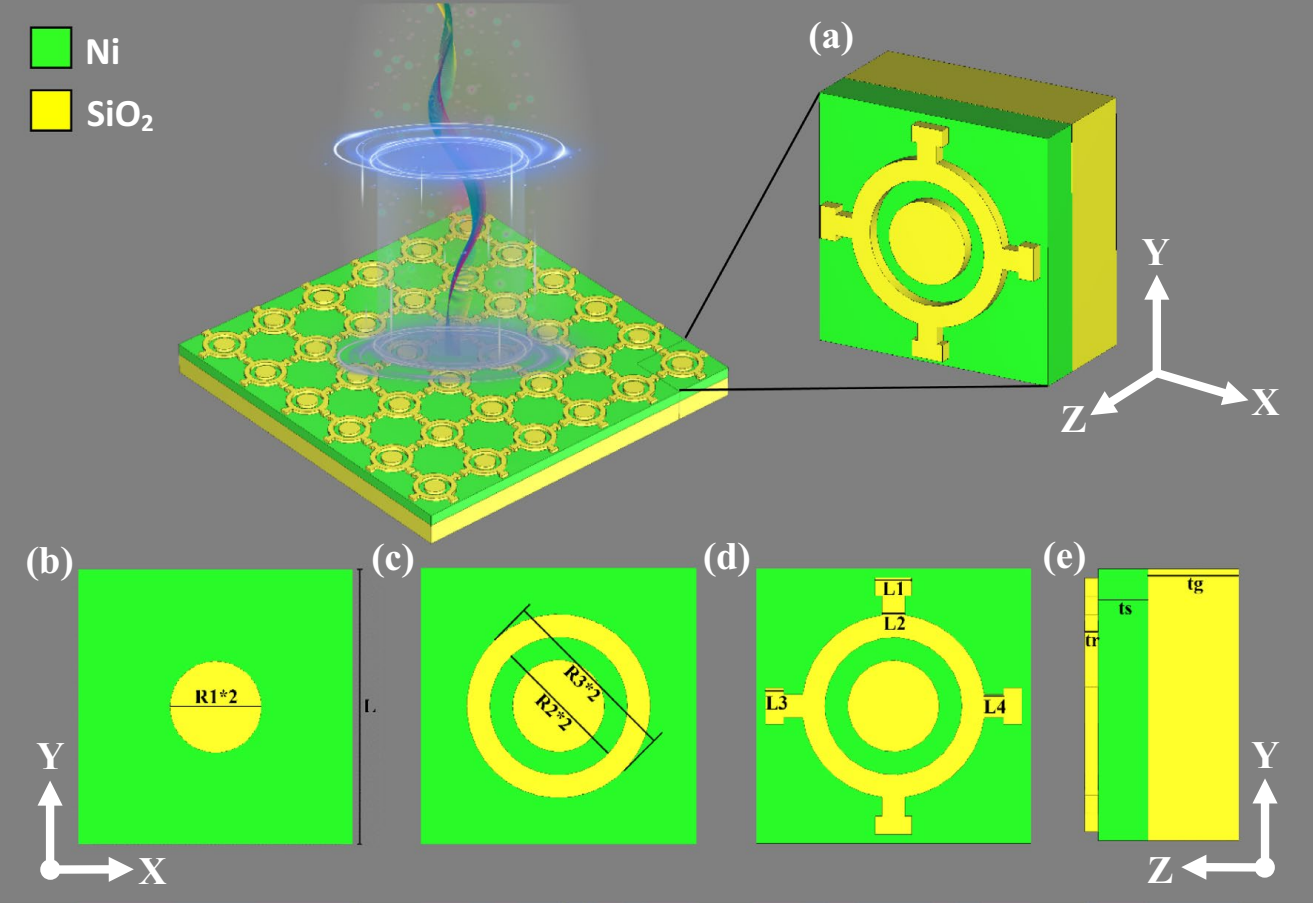
Design evaluation of the unit cell structure (**a**) circular shape added with the radius of R1, (**b**) ring added with inner radius R2 and outer radius R3, (**c**) Proposed unit cell in front view in y–x axis, (**d**) dimensions in side view in y–z axis, and (**e**) proposed unit cell in perspective view.

The physical dimensions of the unit cell should be very accurate and the structure should be symmetric to achieve close-to-unity absorption and insensitive polarization^18^. Figure 1 shows the progressive formation of the unit cell. The final complete design of the structure is depicted in Fig. 1e. The onward and abaft layers are made with Ni-lossy metal, depicted in green, and the SiO_2_ dielectric spacer is depicted in yellow. To design the proposed MIM MMA, two 300 nm-long square boxes were formed, initially with 100 nm and 55 nm thickness, respectively. In order to block the EM wave propagation, the bottom square’s thickness was set to be larger than the skin depth^42^. A circular shape with radius R1 = 50 nm was then added at step 1, pictured in Fig. 1a. Following this, a cylindrical shape with external radius R3 = 100 nm and internal radius R2 = 75 nm was allocated at step 2, as depicted in Fig. 1b. Finally, at step 3, shown in Fig. 1c, four T-shape solids were added. Figure 1c shows the measurements of the T-shape structure. The thickness of the front layer, dielectric layer, and back layer are depicted in Fig. 1d. The total 3D view of the architecture is shown in Fig. 1e. The dimensions of the structure are 300 × 300 × 170 nm^3^. This ultrathin structure is suitable for solar thermophotovoltaics (STPV) cells.

## Results and discussion

### Characteristics of absorption

**Figure 2:**
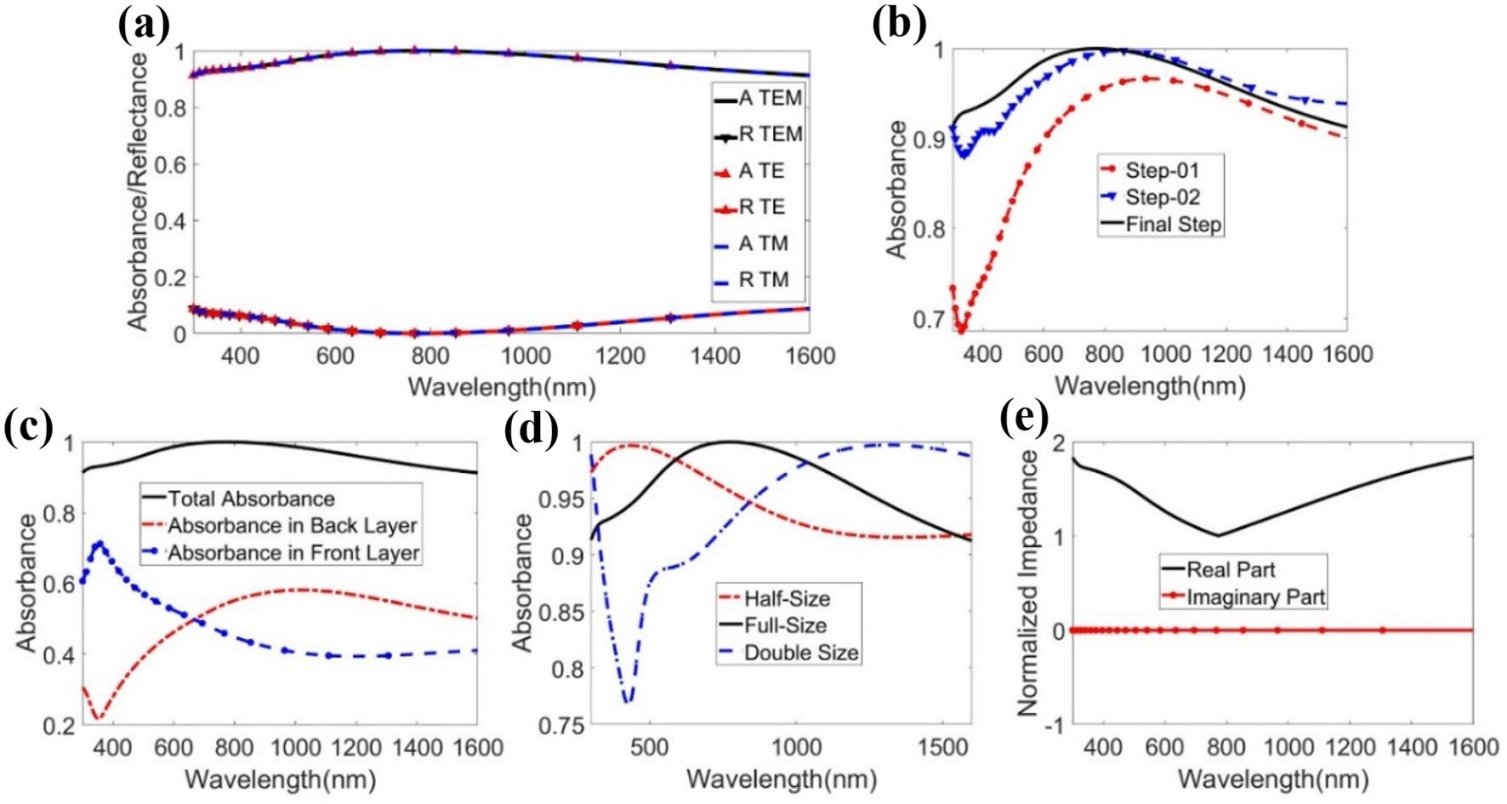
Here, (**a**) absorption in three EM mode TEM, TE, and TM, from 300 to 1600 nm, (**b**) step by step design evaluation absorption, (**c**) absorption in back and front layer, (**d**) absorbance in half and double of the size, (**e**) real and imaginary parts of the.

The performance of the proposed structure based on absorbance characteristics is depicted in Fig. 2a for the wavelength span of 300 nm to 1600 nm, encompassing the UV–visible-NIR domain for TE, TM, and TEM modes. In TEM mode, the proposed unit cell structure holds excellent average absorption: 92.87%, 96.89%, and 97.34% in the UV, visible, and NIR regions, respectively. The average absorption is 95.77% for the whole bandwidth of 1300 nm of the solar spectrum. In TE as well as TM mode, the average absorption is 95.75%—very close to that of TEM mode, thanks to the design’s perfect symmetric nature. The peak absorption of 99.99% is found at 772.82 nm for all three modes in the proposed symmetric unit cell. The average absorptions for edge wavelengths are 91.36% and 91.27%. The absorption level is equal to or above 99% at 621.84–962.12 nm wavelength, indicating that the structure is a near-unity absorber for 340.28 nm bandwidth. The high absorption is the result of high impedance matching, fine coupling capacitance, and inductance. As the structure shows excellent absorption in the UV to NIR region (solar radiation zone), the suggested structure may be efficiently utilized in renewable solar-related energy harvesting^43^.

The absorption characteristics for the design sequence of the proposed structure are shown in Fig. 2b. The design sequence from Fig. 1a–c is designated at step 1, 2, and final step respectively. The average absorption increases sequentially as the design progresses to the final step. The suggested structure consists of two layers: the front layer, in which the metallic Ni resonator thin film and dielectric SiO_2_ film reside, and the metallic Ni ground slab. These two layers’ contribution to absorption characteristics is illustrated in Fig. 2c. As can be seen there, the lower absorption of one layer is compensated by the other layer to achieve excellent absorption as a whole unit cell. The average absorption for the front and back layers is 55.72% and 81.33%, respectively. The reason for this lies in matched impedance of the Ni layer and free space, the lossless characteristics of SiO_2_, the variation of light entrapment at different wavelengths in the SiO_2_ film, and the inductance and capacitance properties of the resonator^15^. The total absorption is the combined contribution of the front and back layers of the suggested unit cell. The proposed design is scaled down to half and scaled up to double in 3 dimensions to observe the absorption characteristics of size variation, as depicted in Fig. 2d. The average absorption for half-size and double-size absorbers are 95.23% and 89.54%, respectively. If the size of the resonator layer is varied, the impedance matching and the structure’s capacitance and inductance values change accordingly, resulting in a shift in the resonant wavelengths. The resonant wavelength is red-shifted when the size of the proposed absorber increases from half to double. The half-size absorber can also be used for absorbing frequencies with a smaller size as it possesses a very high absorbance. As seen in Fig. 2e, the normalized impedance of the proposed MMA possesses an imaginary part of zero at the full wavelength domain. The real component of the normalized impedance becomes one at the resonant wavelength in which maximum absorption occurs. At other wavelengths, the real component of the normalized impedance holds a positive value slightly greater than one. These phenomena aid the absorption properties of the proposed symmetric design due to the high impedance matching the free space^44^.

### Photon mobility for conversion and absorption

**Figure 3:**
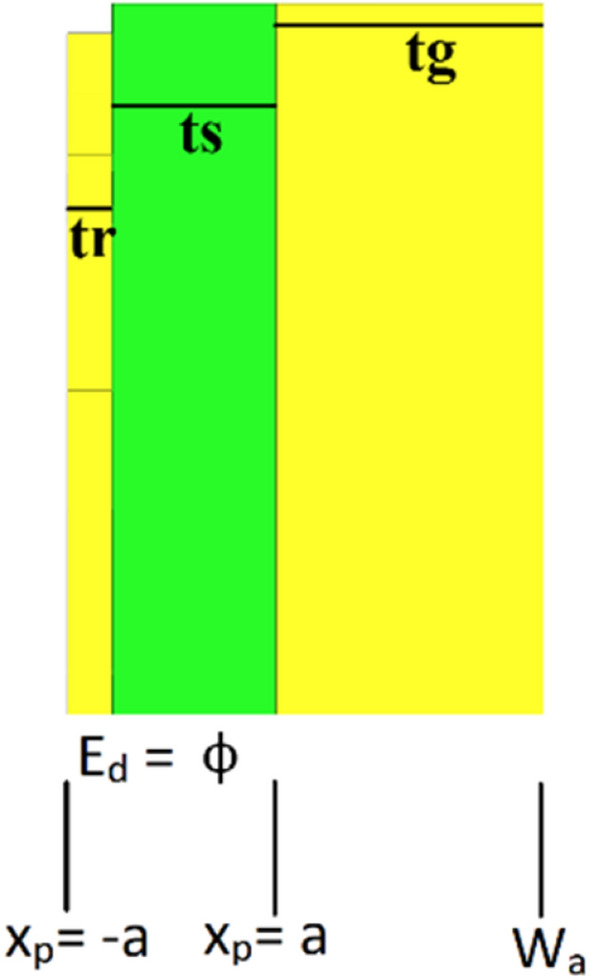
Conceptual layout of the proposed absorber for photon mobility calculation.

Generally, converting incident light into photocurrent is one approach for increasing maximal photon accumulation. The photocurrent is exactly proportional to the intensity of the incident light. There are two phases to this conversion procedure. In the absorbing surface, an electron–hole pair is formed by the photons from the incident sunlight. During energy band escape, some pairs start generating photocurrent, while others end up losing their potential energy. An electron’s energy level increases when a photon is absorbed by a substance. The amount of energy delivered by a photon when it strikes an object is measured by E_p_ = hc/$\uplambda$, where E_p_ is the photon energy, c is the light velocity, $\lambda$ is the wavelength, and h is Planck’s constant. To measure solar irradiance, irradiance density E($\uplambda$) should be proportional to photon energy. The rate of absorption also needs to be consistent in nature because there is a possibility of exposing nonlinearity as the absorption coefficient and wavelength are nonlinear and they reduce the absorption and conversion processes. As a result, mathematical tools may be very useful in such critical optimizations, and transforming a nonlinear function into a linear function might be a viable option. Regression analysis or partial differential equation (PDE) is the most common approach for doing this. Since a photon possesses wave and particle properties, which may be approximated using the Schrodinger equation, in the current study we prefer to use PDE. However, there is a contradicting comment on that. While we model the MMA for solar cells, we go through Nelson's ideal photoconversion criteria^45^. The energy gap in the absorber material distinguishes states that are typically filled from those that are normally empty. In visible wavelength, the incident light with energy E greater than bandgap energy E_g_ is absorbed. Transportation of a photon through a photon tunnel must occur with no loss. When an electromagnetic wave travels through a unit cell, photons excite electrons and the generated potential difference changes, causing the electrostatic potential ($\upphi )$ to vary spanning the whole width of the metal to the dielectric layer. To compute the electron mobility excited by a photon, the boundary condition is demonstrated in Fig. 3, where x_p_ is the position variable along with the thickness of the proposed MMA and the surplus energy over the Fermi-level (0 < E_d_ < $\mathrm{\hslash \omega }$) is denoted by E_d_. Here, $\mathrm{\hslash \omega }$ is the energy equal to if a photon is absorbed and $\mathrm{\hslash }$ is the reduced Planck’s constant.

$${\text{x}}_{{\text{p}}} = - {\text{a }}\;{\text{when}}\;{\text{ Ed}} = 0$$

$${\text{x}}_{{\text{p}}} = {\text{a }}\;{\text{when }}\;{\text{Ed}} = \phi$$

The total area of the substrate across which any photon must travel is denoted by W_a_. Consider a photon passing through the metal via direct transition, where $f(E-\hslash \omega )$ is the primary state and $f(E)$ is the final state. According to Sakhdari et al.^46^, the absorbed photon energy would equal Eq. (1),

1 $$D\left(E,\mathrm{\hslash }\omega \right)=f\left(E-\mathrm{\hslash }\omega \right)g\left(E-\mathrm{\hslash }\omega \right)f\left(E\right)\left(1-g\left(E\right)\right)$$

where $\mathrm{g}\left(\mathrm{E}-\mathrm{\hslash \omega }\right)\mathrm{ and g}\left(\mathrm{E}\right)$ stand for the number of initial electronic states and final electronic states, and $g\left(E\right)=\frac{1}{{exp}^{\frac{(E-{E}_{F})}{KT}+1}}$ the Fermi–Dirac distribution function, assumed to be at room temperature, defines the energy level occupancy probability. It's worth noting that for solar energy harvesting, a junction of metal–semiconductor interface with electrons flowing in both directions is generally necessary, if ${E}_{F}(\mathrm{Fermi energy})<E<{\Phi }_{B}$ (Schottky barrier energy). However, in most cases, the electron is excited by the photon, which may be used to enhance absorption by focusing the photon. The two-dimensional Poisson's equation and the finite difference method are used to compute the amount of photon diffusion and mobility for conversion and absorption. Poisson's equation for charge distribution is

2 $$\frac{{d}^{2}\phi }{d{x}^{2}}=\frac{q(x)}{K{\varepsilon }_{s}} {E}_{d}\,at\,x<0$$

3 $$\frac{{d}^{2}\phi }{d{x}^{2}}=-\frac{q\left(x\right)}{K{\varepsilon }_{s}} {E}_{d}\ at\ x>0$$

where K is the dielectric constant and ${\varepsilon }_{s}$ is the substrate's permittivity. To extract the potential, we solve Eqs. (2) and (3) by integrating at both boundary conditions.

4 $$\phi =\frac{q{E}_{d}}{2{\varepsilon }_{s}}{(x+a)}^{2} for-a<x<0$$

5 $$\phi =\frac{q{E}_{d}}{2{\varepsilon }_{s}}{(x-a)}^{2}+{E}_{d}\ for\ 0<x<a$$

As both photon absorption and conversion should be evaluated in terms of internal quantum efficiency (IQE) as well as external quantum efficiency (EQE), the aforementioned value on electrostatic potential can be utilized to detect photon diffusion after the substrate to the solar cell (EQE). Assuming that the absorbed photons have stimulated q charges, then according to Chen et al.^47^, in quantum conductance, photon tunneling is possible.

$$\psi \left(\overline{r },t\right)={\psi }_{o}\left(\overline{r },t\right)\mathrm{exp}\left[-\frac{i}{h{\int }_{0}^{t}qV({t}^{\mathrm{^{\prime}}})d{t}^{\mathrm{^{\prime}}}}\right]$$

6 $$\psi \left(\overline{r },t\right)={\psi }_{o}\left(\overline{r },t\right)\sum_{n=-\infty }^{n=+\infty }{J}_{n}\left(\frac{q{V}_{\omega }}{h\omega }\right){e}^{-in\omega t}$$

**Figure 4:**
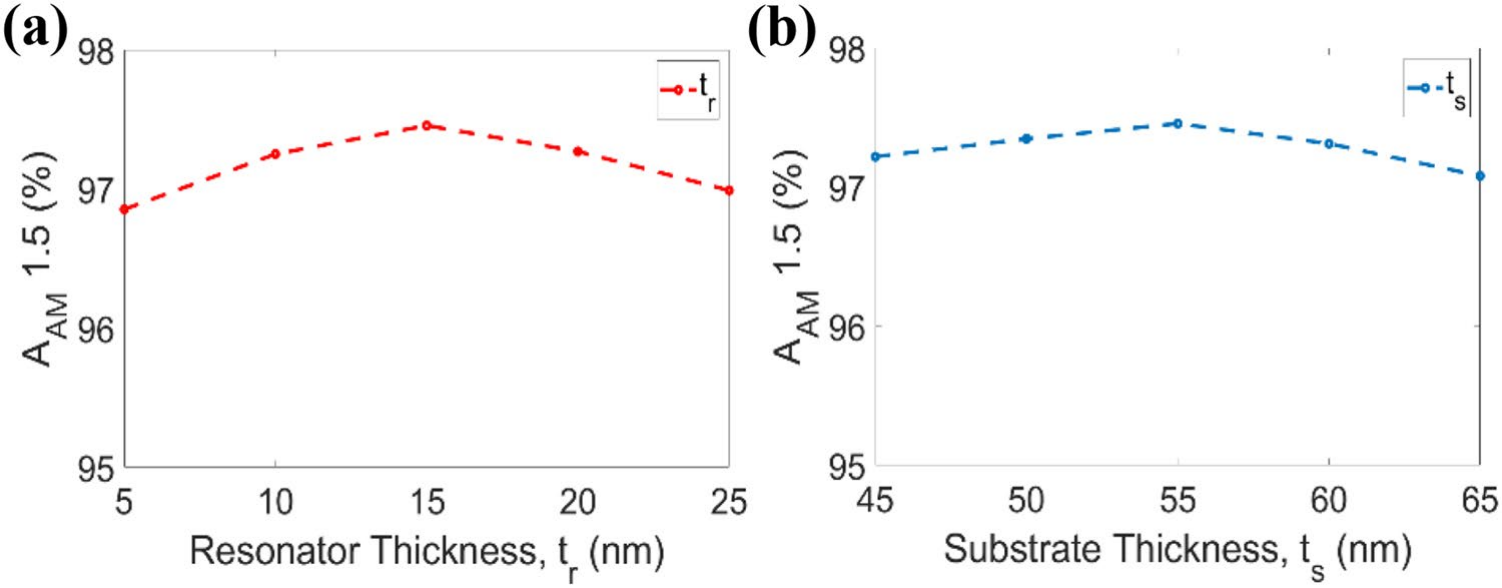
Performance of global standard spectrum in terms of (**a**) resonator thickness tr and (**b**) substrate thickness ts.

The nth-order Bessel function of the first kind is represented by J_n_(x), the unperturbed wave function is represented by ${\psi }_{o}\left(\overline{r },t\right)$, and the time-harmonic dependency is represented by ${e}^{-i\omega t}$, as shown in Eq. (6). The potential energy of electrons can be modulated by this modified wave function at visible wavelength. As a result, the new wave function can trigger a quantum-defined state separated from the ground state by N photons converted or absorbed. The performance of the proposed meta surface-based absorber was compared using the AM 1.5 solar spectrum. We utilized A_AM1.5_ as a benchmark to assess the absorber's absorption capability. Higher A_AM1.5_ values can contribute to higher conversion efficiency^48^. The solar cell's A_AM1.5_ may be calculated using the expression^49^, given in Eq. (7),

7 $${\mathrm{A}}_{\mathrm{AM}1.5}=\frac{{\int }_{{\uplambda }_{\mathrm{min}}}^{{\uplambda }_{\mathrm{max}}}\mathrm{ A}\left(\upomega \right) {\mathrm{I}}_{\mathrm{AM}1.5}\left(\upomega \right)\mathrm{ d\omega }}{{\int }_{{\uplambda }_{\mathrm{min}}}^{{\uplambda }_{\mathrm{max}}} {\mathrm{I}}_{\mathrm{AM}1.5}\left(\upomega \right)\mathrm{ d\omega }}$$

where ${\uplambda }_{\mathrm{max}}$ and ${\uplambda }_{\mathrm{min}}$ define the absorption range of the suggested solar absorber. The photons that are distributed from the sun toward the unit cell are referred to as ${\mathrm{I}}_{\mathrm{AM}1.5}\left(\upomega \right)$. Here, ${\mathrm{I}}_{\mathrm{AM}1.5}\left(\upomega \right)=W(\lambda )/ E(\lambda )$, where $W(\lambda )$ is the solar spectral irradiance and $E(\lambda )$ is the corresponding photon energy. To obtain the number of photons, the standard spectral irradiance distribution ASTM G173-03 was used. ${\mathrm{A}}_{\mathrm{AM}1.5}$ is calculated under the variation of resonator thickness tr and substrate thickness ts, shown in Fig. 4a and b, which evaluates whether the actual parameter is taken into account in the design of the proposed MMA. Ground layer thickness is not taken into consideration for ${\mathrm{A}}_{\mathrm{AM}1.5}$ because its skin depth is much too high to block the propagation of EM waves. In terms of the suggested absorber’s geometrical parameters, the solar spectral irradiance levels are noteworthy, offering great potential for high photon conversion as well as absorption efficiency in solar cells.

### Co-polarization and cross polarization of the proposed design with polarization conversion ratio (PCR)

**Figure 5:**
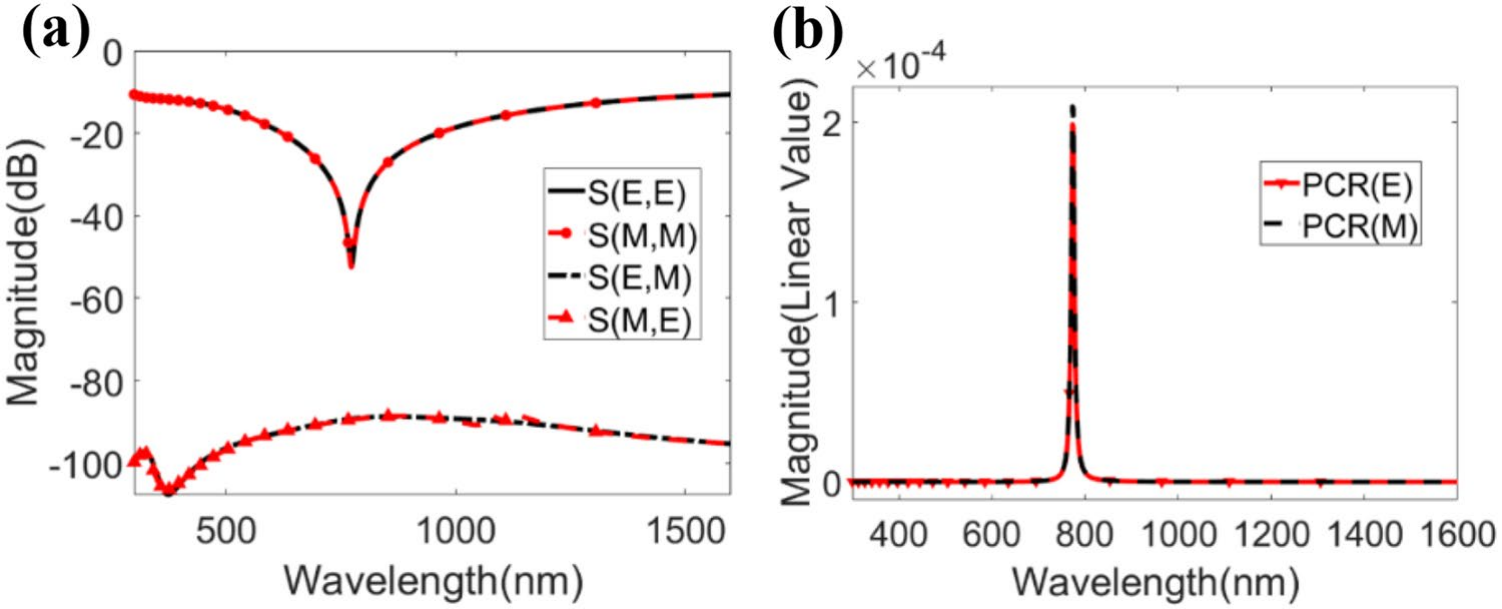
(**a**) Demonstration of co-polarization and cross-polarization, and (**b**) PCR of the structure in both TE and TM mode.

For both TE and TM modes, the suggested MMA does not change the EM wave, and the polarization conversion ratio (PCR) is around zero. The problem that always arises when designing an MMA—the MMA converting polarization rather than perfect absorption — has been reduced, as illustrated in Fig. 5. The graphical depiction of co-polarizations and cross-polarizations is illustrated in Fig. 5a, and the graph of the PCR for both modes is illustrated in Fig. 5b. Co- and cross-polarization were measured using Eqs. (8) and (9), and the PCR was then calculated by using Eqs. (10) and (11):

8 $$\left| {{\text{S}}\left( \omega \right)} \right|^{{2}} = \, \left| {{\text{S}}_{{{\text{E}},{\text{ E}}}} \left( \omega \right)} \right|^{{2}} + \, \left| {{\text{S}}_{{{\text{E}},{\text{ M}}}} \left( \omega \right)} \right|^{{2}} = {\text{ R}}_{{1}}^{{2}} + {\text{ T}}_{{1}}^{{2}}$$

9 $$\left| {{\text{S}}\left( \omega \right)} \right|^{{2}} = \, \left| {{\text{S}}_{{{\text{M}},{\text{ M}}}} \left( \omega \right)} \right|^{{2}} + \, \left| {{\text{S}}_{{{\text{M}},{\text{ E}}(}} \omega )} \right|^{{2}} = {\text{ R}}_{{2}}^{{2}} + {\text{ T}}_{{2}}^{{2}}$$

10 $${\text{PCR}}_{{\text{E}}} = {\text{ T}}_{{1}}^{{2}} / \, \left( {{\text{R}}_{{1}}^{{2}} + {\text{ T}}_{{1}}^{{2}} } \right)$$

11 $${\text{PCR}}_{{\text{M}}} = {\text{ T}}_{{2}}^{{2}} / \, \left( {{\text{R}}_{{2}}^{{2}} + {\text{ T}}_{{2}}^{{2}} } \right)$$

where |S_E, E_ (ω)|^2^  = |S_M, M_(ω)|^2^ = R_1_^2^ = R_2_^2^ = reflectivity of X-polarization and |S_E, M_(ω)|2 = |S_M, E_(ω)|^2^ = T_1_^2^ = T_2_^2^ = reflectivity of Y-polarization. The PCR can be expressed as the ratio of X-polarized reflectivity over the total reflectivity. Figure 5b shows that the PCR value for the transverse electric (PCR_E_) and transverse magnetic (PCR_M_) modes are negligible, that nullify the matter of PCR factors. The main reason behind this factor is T_1,_ which has a relatively modest quantity.

### Numerical verification

**Figure 6:**
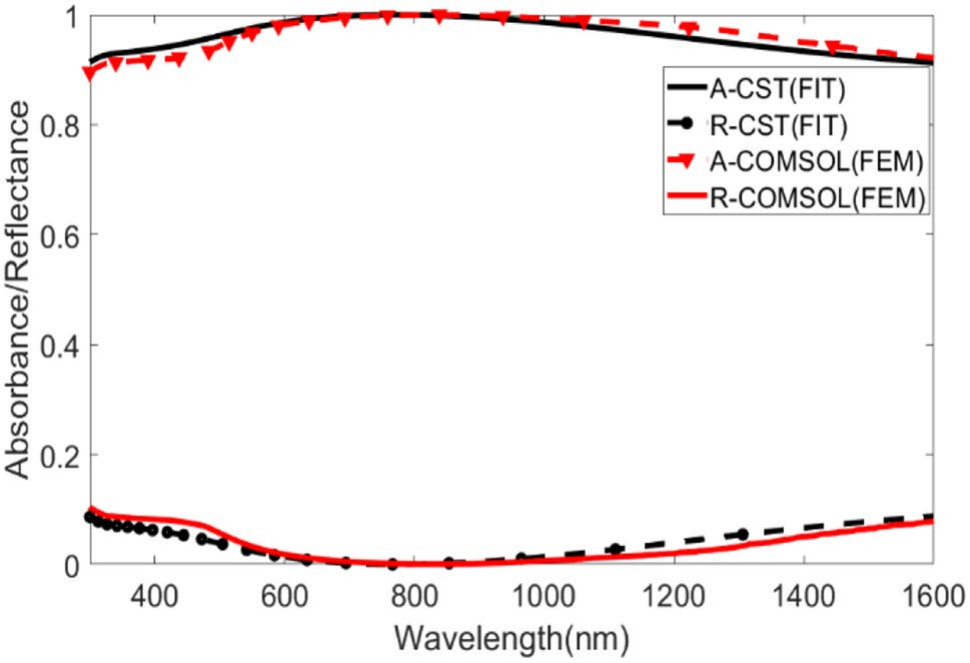
Visualization of the numerical verification of the design with FEM method in COMSOL Multiphysics simulation software and FIT method in CST MWS simulation software of the proposed design in normal incidence with TEM mode. Insets: Demonstration of simulation setup in both simulation software.

The integrity of the proposed MMA unit cell design underwent verification by COMSOL Multiphysics Simulation software, as illustrated in Fig. 6. CST, the primary simulation software in this study, uses the finite integration technique (FIT), and COMSOL, the secondary simulation software, conducts finite element method (FEM) for numerical computation.

In COMSOL, the proposed structure exhibits 96.26% average absorption with a resonant wavelength of 831.32 nm. The offset in the average absorption is 0.49% from the CST output, and the resonant wavelength is shifted 58.5 nm to the right. The peak absorption is 99.96% at the resonant wavelength in COMSOL, which is 0.03% lower than the primary result. This small variation occurs due to the meshing in the COMSOL software affecting the output result^50^. A moderate mesh is selected in the COMSOL design due to limited available processing power. However, as seen in Fig. 6, the shapes of the absorbance and reflectance curves for both numerical outputs are almost the same. The proposed design in both numerical approaches demonstrate high average absorption in the UV–Vis–NIR domain, with the near-unity peak at the resonant wavelength.

### Incident angle stability

**Figure 7:**
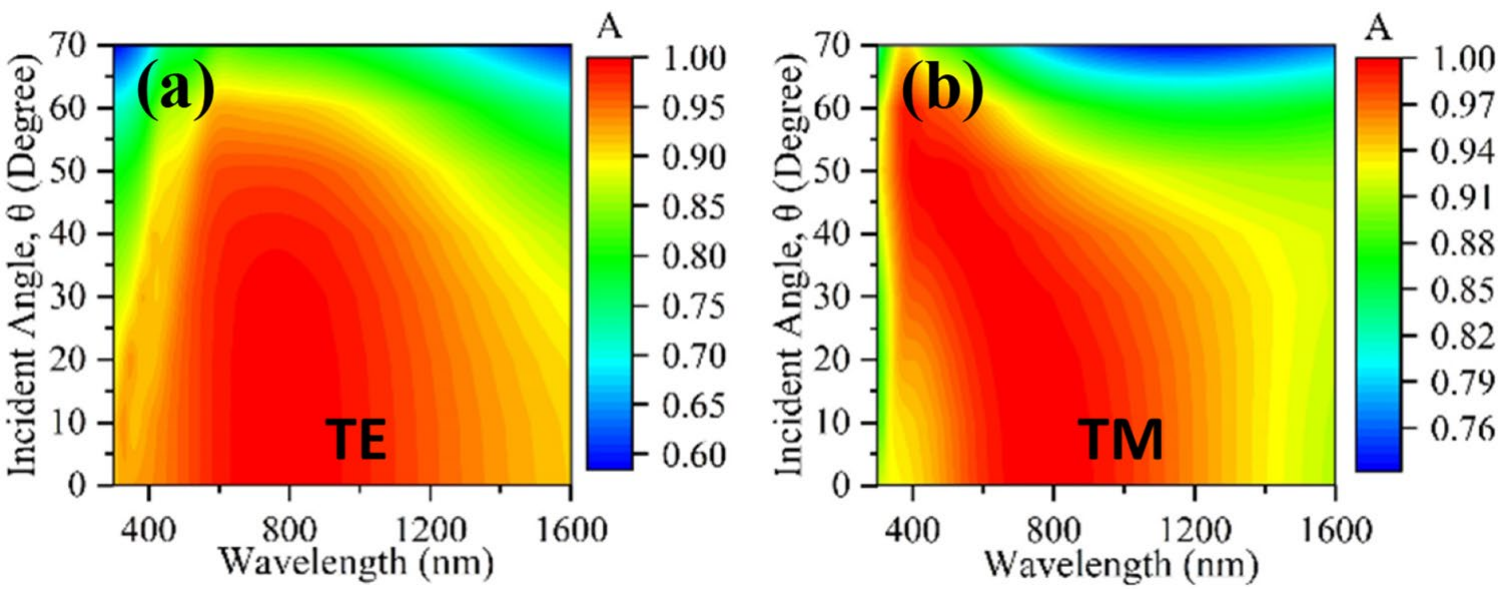
Demonstration of (**a**) incident angle of TE mode from 0° to 70°, and (**b**) incident angle of TM mode from 0° to 70°.

Figure 7a and b shows the variation of the incident angle from 0° to 70°, for both TE and TM modes. With increasing incidence angles, the average absorption falls, and it has long been recognized that a higher angle of incidence means longer path length and lesser impact of coupling. The structure's electromagnetic dipolar resonance decreases as a result of the reduced coupling effect, therefore reducing the dielectric layer's ability to confine waves^22^. Absorption is calculated using specular reflection since the simulation shows that when the incident angle deviates 10° from the perpendicular direction, the reflected field strength drops by around 10 dB, implying that the reflected energy is mostly distributed in that direction. In this study, the resonator consists of a circular ring in the center that enables wide-angle insensitivity. The ring's circumference is where the electric fields are most intense, as illustrated in Fig. 11a–d, and its parameters have been optimized to minimize angle sensitivity^51–53^. As a result, the reported absorber is an excellent option as a polarization-insensitive broadband metamaterial solar absorber with incidence angle stability.

### Parametric sweep

**Figure 8:**
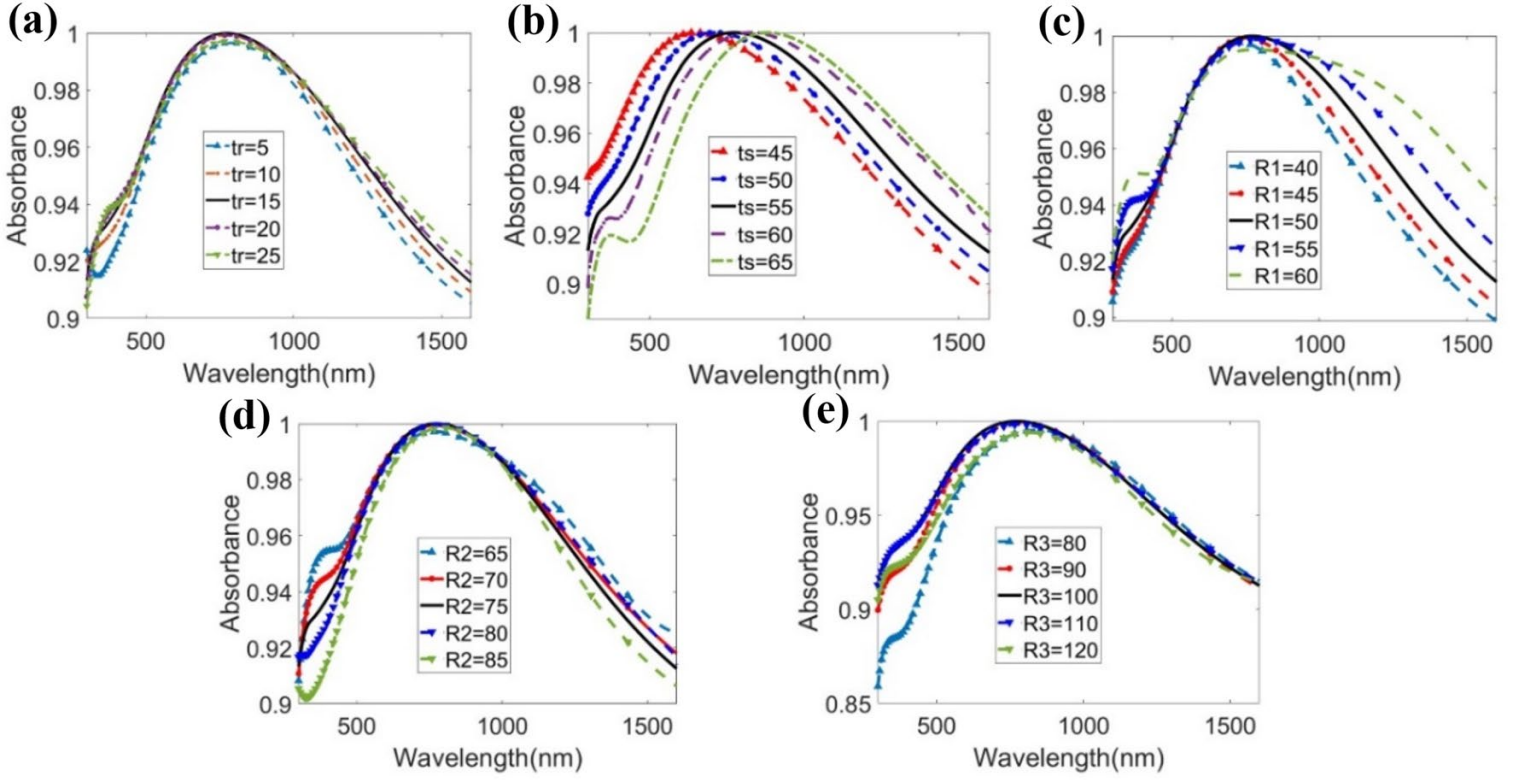
Parametric analysis for parameter (**a**) thickness of the resonator tr, (**b**) substrate thickness ts, (**c**) radius of the central cylinder in resonator R1, (**d**) inner radius of ring shape in resonator R2, (**e**) outer radius of ring shape in resonator R3.

The effects in the absorption properties of the important geometric parameter variation are illustrated in Fig. 8. The parameter tr is swept from 5 to 25 nm with a step of 5 nm, as shown in Fig. 8a. The average absorption is found to be in the range of 94.98% to 95.77%. The average absorption is the highest at the proposed tr value of 15 nm as the free space impedance is perfectly matched with the metamaterial unit cell and the resonance condition also occurs. Impedance match highly depends on the capacitance created by the metal plane and metal resonator. Due to Ni’s good metallic property and the structure ruptured by the EM waves, the metal resonator creates good capacitance with the back metal plane, which is inversely proportional to its thickness. Hence, the resonant wavelength shifts slightly.

The parameter ts is swept from 45 to 65 nm with a step of 5 nm, as depicted in Fig. 8b. The resonant wavelength is red-shifted as the dielectric width increases. By altering the dielectric thickness, the capacitive and inductive effects are altered, and the average absorption therefore changes as well. Again, the variation in the current density in the ground slab is affected by the dielectric spacer thickness, which causes the red-shifting of the resonant wavelengths^54^. By changing the dielectric thickness, various specific wavelengths can be detected by the proposed cell structure, as the resonance is dependent on the ts value as seen from Fig. 8b.

Different radius parameters, denoted as R1, R2, and R3 in Table 1, are swept to analyze the effect in the absorption properties, as shown in Fig. 8c–e. The values of R1, R2, and R3 jointly affect the impedance matching of the resonator to the free space as well as the structure’s capacitance and inductance. The effects of that property variation result in the alteration of the average absorption as well as the shifting of the resonant wavelength. The average absorption gradually increases by very small increments of R1 from 40 to 60 nm. However, the volume of the Ni metal layer will increase as R1 increases. Thus, R1 is set to 50 nm in the proposed structure to achieve high average absorption while maintaining a smaller size. The increase in R2 from 65 to 85 nm causes a small redshift in the wavelength of resonance because of the variation in the capacitance and inductance in the absorber front layer. The peak absorption is highest at R2 = 75 nm, with a value of 99.999%, the highest among all measurements taken for the proposed unit cell. From the R3 parameter sweep analysis, the highest average absorption occurs at R3 = 100 nm. The highest peak absorption within R3 also occurs at this value. Thus, this radius was selected for the suggested MMA structure.

**Table 1:** Parameter List of the proposed model.

Parameters	L	R1	R2	R3	L1	L2	L3	L4	tr	ts	tg
Value (nm)	300	50	75	100	40	20.78	20	25	15	55	100

### Absorption comparison with different types of metals and di-electric

**Figure 9:**
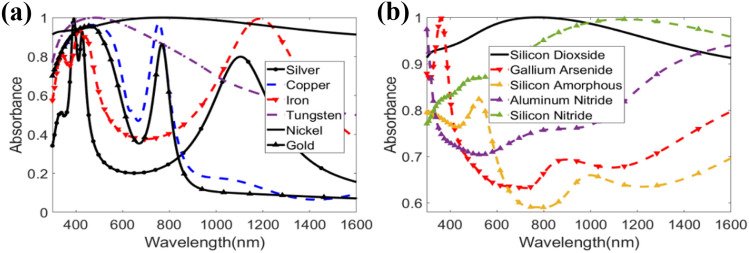
Absorption with different (**a**) metal layers, and (**b**) dielectric substances.

To ensure the performance of the absorber, the suggested MMA was tested with a variety of metals and dielectrics other than Ni and SiO_2_. Without changing the dielectric, the absorption response of silver (Ag), copper (Cu), iron (Fe), tungsten (W), and gold (Au) metals is shown in Fig. 9a. If the top and bottom metal layers are replaced with Cu, Fe, Ag, or Au, the MMA can be utilized as a dual-band or multiband absorber, as shown in the figure. With a mean absorption of 96.7%, an MMA with W in this design has good absorption across the full visible range (380 nm to 780 nm). However, in this case, we picked Ni since a solar energy harvester contains 44.7% light waves, 6.6% UV radiation, and 48.7% infrared^55^, and Ni has the whole UV–Vis–IR absorption with an average of 95.78%. It is known that the metamaterial unit cell physical properties of effective permittivity (ε_r,eff_) and effective permeability (μ_r,eff_) are dependent on the combination of the metallic resonator’s shape, material, and dimensions^56^. These physical properties contribute to the impedance of the proposed metamaterial structure^57^. For the proposed hexagonal resonator and dimensions, Ni shows advantageous values of ε_r,eff_ and μ_r,eff_ such that the impedance of the suggested structure matches with the free space impedance. Thus, the absorbance is much higher for Ni than other metals for the proposed metamaterial structure system. The numerical findings for several dielectrics such as silicon dioxide (SiO_2_), gallium arsenide (GaAs), amorphous silicon (A-Si), aluminum nitride (AiN), and silicon nitride (Si_3_N_4_) with the same Ni metal for the top and bottom layers are shown in Fig. 9b. The peak wavelength shifted in accordance with the refractive index of the dielectric^42^. GaAs can be used as a dielectric with the proposed design for sensing purposes at 300 nm to 600 nm. A-Si, AiN, and Si_3_N_4_ can also be used as solar absorbers with the proposed MMA as they have mean absorption rates of 72.35%, 76.93%, and 87.95%, respectively. However, they are not efficient as SiO_2._ The use of SiO_2_ as a substrate material relates to the transient permittivity characteristics and optical threshold breakdown relying on electron number density. Silica's visible spectrum dielectric response has a relatively negative real part of permittivity in comparison to Si rather than a high imaginary part of the dielectric constant^41^. As a result, at the breakdown condition, the real component of permittivity becomes zero, bringing the propagating wave closer to the nature of an evanescent wave. Furthermore, the refractive index dispersion relation for silica is highly birefringent. This anisotropic characteristic lends itself well to changing polarization and propagation control via the substrate. As a result, SiO_2_ meets the criteria. Its resonant qualities also help in the design's impedance matching. The dielectric film is known to alter inductance and coupling capacitance related to the ground layer and resonator. SiO_2_ aids the suggested construction by keeping the inductance and coupling capacitance at a favorable value. As a result of the combined impacts of the two materials in the construction, the bandwidth of maximum absorbance increases in the optical spectrum.

### Mechanical stress

During production and imposition of the MMA, several mechanical deformities can be induced^58,59^. Hence it is necessary to analyze the behavior of the MMA in the case of aberrations. The purpose is to develop a near-unity MMA, such that the electro-optical features of the proposed structure have been analyzed in accordance with the mechanical deformation effects. The purpose of checking the absorption properties is to obtain a satisfactory level, mainly when two kinds of mechanically deformed bending—concave (positive) and convex (negative)—have been applied. Convex deformation is caused by tensile tension, while concave deformation is caused by compression. With non-uniform mechanical stress, the entire structure was bent from − 10° to 10° in increments of 2°. No mechanical stress is applied in the case of 0°. The maximum vM stress was also computed using^60–62^ for numerical simulation.

#### Absorption response under convex bending

**Figure 10:**
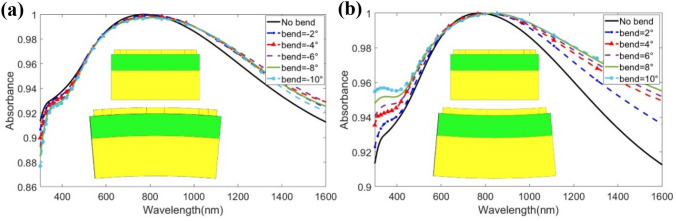
Absorption phenomenon of the proposed structure under (**a**) negative/convex bending from 0° to 10°, and (**b**) positive/concave bending from 0° to 10°. Insets: (**a**) representation of negative bending with *θ* degree, and (**b**) positive bending with *θ* degree.

Tensile stress causes convex deformation in the unit cell. The numerical results for convex bending are shown in Fig. 10a. When the bending moment is extended from − 10° to 0° (no bending) in 2° increments, a small redshift occurs. The illustration shows that at an extreme bending angle of − 10°, minimum absorption occurs, at 83.7%. With increasing convex bending, peak absorption and average absorption both decrease slightly. This is due to changes in coupling capacitance (C) and inductance (L) since when C and L change, the resonant frequency changes as well. Concurrently, the average absorption at − 10°, − 8°, − 6°, − 4°, and − 2° is 94.4%, 94.77%, 95.26%, 95.58%, and 95.78%, respectively. Therefore, the proposed MMA has a minimum 94.4% absorption under convex deformation, making it ideal for use as a solar absorber. Table 2 shows the associated vM stress for the stated bending angles.

**Table 2:** Bending angle in degrees with maximum vM stress in GPa for proposed MA.

Negative/convex bending						Positive/concave bending					
Bending angle (degree)	− 2	− 4	− 6	− 8	− 10	Bending angle (degree)	2	4	6	8	10
Maximum vM stress (GPa)	1.47	2.83	5.52	8.69	18.53	Maximum vM stress (GPa)	1.47	2.83	5.52	8.69	18.53

#### Absorption response under concave bending

Unanticipated compression during the production or application process can cause concave distortion in the MMA. The maximal vM stress rises as the concave moment increases in the unit cell, as depicted in Table 2. The numerical results under non-uniform mechanical load in the unit cell are shown in Fig. 10b. The absorption rates increase when the bending moment increases, as seen in the image. This is due to coupling capacitance and inductance resonance. At 2°, 4°, 6°, 8°, and 10°, the mean absorption is 95.78%, 96.65%, 97.26%, 97.8%, and 98.2%, respectively. At a 10° extreme bending moment, this MMA shows an excellent mean absorption of 98.2%. As a result, the proposed MMA may be expected to perform admirably in the field of solar thermal harvesting.

### E-field, H-field, and surface current density

**Figure 11:**
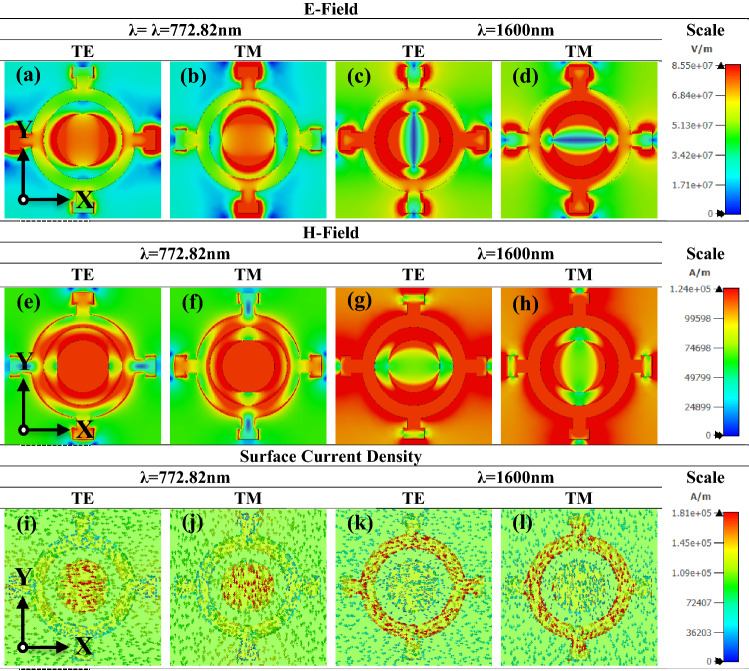
Demonstration of (**a**–**d**) E-field of the proposed model for TE and TM mode in 772.82 nm (max point) and 1600 nm (min point) with scale bar (V/nm) in y-x axis cross sectional view, (**e**–**h**) H-field of the proposed model for TE and TM mode in 772.82 nm (max point) and 1600 nm (min point) with scale bar in A/m in y–x axis cross sectional view, and (**i**–**l**) surface current density of the proposed absorber for TE and TM mode in 772.82 nm (max point) and 1600 nm (min point) with scale bar (A/m) in y–x axis cross sectional view.

To investigate the absorption mechanism and RF response of the proposed absorber, the electric field (EF), magnetic field (HF), and surface current density (SCD) are discussed below, with an associated presentation in Fig. 11a–l for the peak (99.999%) and low (91.27%) absorption points of 772.82 nm and 1600 nm, respectively. The figure shows that the EM field is resonantly focused and intensified at some regions of the absorber at a given wavelength, and that the field distribution changes along with the polarization mode for the EF, HF, and SCD. A dipolar magnetic moment formed for back-to-back reflection at the interface of the top and back metal^63,64^. As stated earlier, the back metal layer blocks the transmission of incident EM wave. This enhances dielectric resonance—which serves as a primary cause of broadband absorption—and induces a further surface plasmon that boosts EF growth. As MM is different from conventional materials, the conventional EM field distribution cannot satisfy the MMs. EF and HF can be described as D_avg_ = ε_eff_ε_0_E_avg_ and B_avg_ = μ_eff_μH_avg_, respectively, where D_avg_ represents the average electric flux density, ε_eff_ represents the effective permittivity of the medium, ε_0_ the free space permittivity, E_avg_ the average EF intensity, B_avg_ the average magnetic flux density, µ_eff_ the medium's effective permeability, µ_0_ free space permeability, and H_avg_ the average HF density. Taking both formulas into account, quantifying the flux densities in Maxwell's equation (integral form) can be described as Eq. (12):

12 $$\int\limits_{C} {E \cdot dI} = 0 - \frac{\partial }{\partial t}\iint\limits_{S} {B \cdot dS}\;\;{\text{and}}\;\;\;\int\limits_{C} {H \cdot dI} = 0 + \frac{\partial }{\partial t}\iint\limits_{S} {D \cdot dS}$$

Around the surface, the parameters are treated as E = EF intensity, D = electric flux density, H = HF density, and B = magnetic flux density. When an electromagnetic wave travels through a unit cell, the integral function is defined along its surface or when the HF indicates non-uniform variation with quick changes. In a uniform field distribution, permittivity achieves unity; however, in this case, H and B are asymmetric and non-uniform, as is the consequent EM wave propagation. In this article, the dielectric properties (ε, μ) of the nanostructure resonator are obtained using the standard approach of gathering material parameters from transmission/reflection data^65,66^. For both TE and TM modes, the EF is substantially contained in the dielectric spacer (SiO_2_), inducing the resonant dipolar moment. This helps to absorb the EM wave. The E-field is shown in Fig. 11a–d for both TE and TM polarization for two different wavelengths, as mentioned earlier. It should be noted that the EM propagation changes with the shift in polarization mode. The dispersed EF is located predominantly at the cell center. Figure 11e–h shows the HF for both TE and TM modes with prescribed wavelength, which follows the same distribution as the EF since the structure has a perfect symmetric geometry. The anti-parallel circulating current induced at the interface of the resonator layer and base metal layer generates a magnetic moment that is immersed on the dielectric layer, thus reducing the reflections^67,68^. A higher HF is induced due to the localized surface plasmons resonance along the whole working spectrum. This dispersed HF pattern implies that the planned meta surface traps the EM incidence wave, which appropriately aids wideband absorption. Figure 11i–l shows the circulating surface current and significant distributed surface charge for both TE and TM modes for resonance wavelength **λ = **772.82 nm and the lower absorption point of 1600 nm for the focused working spectrum. The most distributed surface is situated at resonance wavelength 772.82 nm for both TE and TM polarization. Comparatively, a low-surface charge can be found at the other end of the spectrum (300 nm to 1600 nm). The consistent anti-parallel circulation of the surface charge explains why the structure creates a large electromagnetic field in the dielectric layer^69^.

### Comparative study of the proposed design and other designs with similar features and relatively close bandwidth

An MMA with good average absorption, polarization insensitivity, incident angle stability, and design compactness in the UV to NIR region is hard to find. Table 3 provides a comparative look with other similar broadband absorbers to explore the acceptability of the proposed absorber. The proposed absorber provides a mean absorption of 95.77%, with an extensive bandwidth of 1300 nm in the region of UV to NIR. Moreover, it shows a near-unity absorption of 99% over a bandwidth of 340 nm. In terms of polarization and incident angle of the electromagnetic wave, the proposed absorber is polarization insensitive from 0° to 90° and up to 70° incident angle stable, with a considerable rate of average absorption. It can tolerate mechanical bending in either the concave or convex position up to 10°, which differs from the other proposed absorber. With temperature stability being a key factor for maintaining the absorber’s performance, Ni and SiO_2_ are used for their higher thermal stability. In short, the proposed absorber’s compact symmetric design, thermal stability, ability to bear mechanical stress, cost-effectiveness due to compact size, ultra-broadband absorption with polarization insensitivity, and incident angle stability enhance its acceptability when compared with other similar absorbers.

**Table 3:** Comparison table of the proposed model with previously published models.

Range (nm)	Number of layer	Dimensions l × w × h (nm^3^)	Materials	Polarization-insensitivity and Angular stability for up to 70% absorption	Absorption level	Average absorption (%)	Peak absorption (%)	Ref.
300–2000	Three	400 × 400 × 825	LiTaO_3_, Au	Yes, $\theta \le 45^\circ$	N/A	76.35	99.95	^70^
485–1492	Three	400 × 400 × 230	Ti, SiO_2_	Yes, $\theta \le 45^\circ$	Above 90%	91.4	97.5	^31^
200–900	Three	300 × 300 × 80	W, SiC, SiO_2_	Yes, $\theta \le 60^\circ$	Above 90%	95	99	^71^
360–1624	Four	360 × 360 × 330	TiN, SiO_2_, TiO_2_	Yes, $\theta \le 45^\circ$	Above 90%	95.68	96.9	^72^
700–1934	Three	1000 × 1000 × 310	Au, SiO_2_	N/A	Above 40%	80.24	96.40	^32^
405–1505	Four	380 × 380 × 415	SiO_2_, Ti, MgF_2_, Al	Yes, $\theta \le 60^\circ$	Above 90%	95.14	99.9	^73^
456–1832	Three	400 × 400 × 295	Ti, SiO_2_	Yes, $\theta \le 60^\circ$	Above 90%	94.6	97.7	^74^
167–1926	Four	500 × 500 × 450	Ti, W, SiO_2_, Au	Yes, $\theta \le 45^\circ$	Above 90%	92.7	99.9	^35^
500–1800	Three	1000 × 1000 × 320	Ti, Al_2_O_3_, W	Yes, $\theta \le 60^\circ$	Above 85%	94	99.99	^33^
300–1600	Three	300 × 300 × 170	Ni, SiO_2_	Yes, $\theta \le 70^\circ$	Above 91.27%	95.77	99.99	[Proposed]

## Conclusion

In summary, the proposed three-layer (Ni-SiO_2_-Ni) MMA unit cell structure is numerically investigated to determine its suitability as a solar energy harvester. It shows a near-unity broadband absorption throughout the wide solar spectrum of 300 nm to 1600 nm, encompassing the UV–Vis–NIR region. The structure is bendable under mechanical stress and shows good absorption under convex as well as concave bending. The universal AM 1.5 solar spectrum performance is applied to the proposed MMA to analyze the photon trapping characteristics of the unit cell. The excellent results suggest that the MMA proposed in this article can be used in a solar cell for any complex electromagnetic environment and at any oblique incident angle for efficient solar energy absorption.

## Methodology

### Simulation setup

In the case of transverse electromagnetic (TEM) mode setting, two periodic boundary conditions, perfect electric conductor (PEC) and perfect magnetic conductor (PMC) were set on the y–z and x–z planes, respectively, while the operational wave propagates from the negative x–y plane through the waveguide port. Transverse electric (TE) and transverse magnetic (TM) polarization modes were used with a master and slave boundary condition on the y–z and x–z planes, where the flouquet port was positioned on the x–y plane, to study the behavior of the suggested absorber for linearly-polarized plane wave (LPPW) spectrum. On the top layer of the unit cell, LPPW wide-spectrum is incident. In CST Microwave-studio (CST MWS), the design was simulated using the finite integral technique (FIT). For numerical validation, the finite element method (FEM) is employed in conjunction with the COMSOL Multiphysics software.

### Calculation of absorption

The abovementioned periodic boundary condition was applied to the unit cell in the x- and y-direction, where the impedance matching the topmost surface of the absorber was kept open in the z-direction to obtain S-parameters that help to calculate the absorption of the suggested absorber using Eq. (13),

13 $$A\left(\omega \right)=1-R\left(\omega \right)-T\left(\omega \right)$$

where $A\left(\omega \right)$ represents the absorption, $R\left(\omega \right)$ the reflection, and $T\left(\omega \right)$ the transmission of the incident electromagnetic waves. Here, reflection and transmission are related to the reflection coefficient and transmission coefficient of the S-parameters.$R\left(\omega \right)={\left|{S}_{11}(\omega )\right|}^{2}$ and $T\left(\omega \right)={\left|{S}_{21}(\omega )\right|}^{2}$. As the bottom layer of the proposed absorber is made of Ni and must be thick enough to block the incident wave penetration, the skin depth of Ni is much higher to block wave propagation through the bottom layer. Skin depth can be calculated by the established equation $\updelta =\sqrt{2\uprho /2\mathrm{\pi f}{\upmu }_{\mathrm{r}}{\upmu }_{\mathrm{o}}}$^42^, where $\uprho$, f, ${\upmu }_{\mathrm{r}},{\mathrm{and \mu }}_{\mathrm{o}}$ represent Ni’s resistivity, frequency, relative permeability, and permeability in vacuum, respectively. Therefore, it can be said that $T\left(\omega \right)={\left|{S}_{21}(\omega )\right|}^{2}=0$. Equation (13) can be rewritten as Eq. (14),

14 $$A\left(\omega \right)=1-R\left(\omega \right)$$

Equation (2) enhances the absorption of the MMA, but performance might be improvised by keeping the reflection coefficient ${S}_{11}(\omega )$ as small as possible. This coefficient will be at a minimum if the impedance of the refractory film of the proposed absorber matches the free space impedance $120\pi \mathrm{or} 376.76\Omega$. Refractory layer impedance depends on the relative permeability and permittivity and may be computed by applying the Nicolson–Ross–Weir (NRW) equation^75^. A high near-field coupling can be achieved between the bottom layer and the Ni-based resonator array. When the coupling is considered, the theoretical results obtained from the interference model match numerical simulations rather well. In addition to the null transmission of the ground plane, it functions by free space impedance matching because of destructive reflection interference. The suggested MMA is lossy high impedance surfaces with significant absorption. The geometry-based surface with exact EF and HF charge distribution lends itself to perfect absorption.
